# Drug assumption and awareness about adverse drug reactions. The right to know. The case of the bone-modyfing agents: a systematic review

**DOI:** 10.3389/froh.2024.1441601

**Published:** 2024-08-01

**Authors:** Rodolfo Mauceri, Sonia Arduini, Martina Coppini, Monica Bazzano, Isabel Trujillo, Giuseppina Campisi

**Affiliations:** ^1^ Unit of Oral Medicine and Dentistry for Frail Patients, Department of Rehabilitation, Fragility and Continuity of Care, University Hospital Palermo, Palermo, Italy; ^2^ Department of Me.Pre.C.C., University of Palermo, Palermo, Italy; ^3^ Department of Law, University of Palermo, Palermo, Italy; ^4^ Department of BIOMORF, University of Messina, Messina, Italy

**Keywords:** awareness, knowledge, patients, bisphosphonate-associated osteonecrosis of the jaw, osteonecrosis of the jaw, medication-related osteonecrosis of the jaw, MRONJ, ONJ

## Abstract

**Introduction:**

Medication-related osteonecrosis of the Jaw (MRONJ) is an adverse drug reaction that affects the mandible and maxilla of patients exposed to BMA and AA therapies, causing the progressive destruction and death of bone. To date, oral health preventive measures remain the most effective strategy to reduce MRONJ incidence, and, in this sense, the major goal is to diagnose, treat, and eradicate any oral diseases that could compromise oral health. The present systematic review aims to investigate the awareness of MRONJ among patients assuming BMAs.

**Methods:**

A systematic literature search was performed, selecting studies that concern the awareness of patients of the risk of MRONJ.

**Results:**

Six studies were included in this review. In total, 483 patients were evaluated. Of the 483 included patients, 391 were not aware of the possibility of MRONJ onset (391/483, 81%) and 92 were aware of it (92/483, 19%).

**Discussion:**

The problem of patient's lack of awareness with respect to MRONJ risk presents different layers of complexity (“what?”, “who?”, “where?”, “when?” and “why?”). Among its causal factors, there are an inadequate level of communication with patients and the lack of collaboration between healthcare professionals, which is related to an individualistic view of liability and deontological duties. MRONJ is a drug adverse reaction that can greatly affect the quality of life of patients if not promptly diagnosed and treated. Therefore, patients must be fully aware of the risks of adverse and the importance of preventive measures, which imply effective and exhaustive communication by each member of the multidisciplinary team. Effective teamwork and collaborative care should be promoted to positively impact patients' awareness.

## 1 Introduction

Medication-related osteonecrosis of the Jaw (MRONJ) has been defined as an “adverse drug reaction described as the progressive destruction and death of bone that affects the mandible and maxilla of patients exposed to the treatment with medications known to increase the risk of disease, in the absence of a previous radiation treatment” (1).

Medications known to increase the risk of MRONJ are bone-modifying agents (BMA) and/or anti-angiogenic (AA) molecules (2).

BMA and AA therapies are mainly prescribed to four categories of patients (1):

• Cancer patients with bone metastases or myeloma patients; generally receiving high dose (HD) BMAs often associated with other agents (e.g., chemotherapy, radiotherapy, endocrine therapy) (3, 4)
• Patients suffering from osteoporosis and other non-malignant diseases receiving BMAs with different regimens (5)
• Patients affected by breast cancer (BC) or prostate cancer (PC) suffering from osteoporosis without bone metastases receiving low-dose (LD) BMAs to limit the risk of non-metastatic bone fractures (due to Cancer Treatment-Induced Bone Loss) (6)
• Patients with Giant Cell Tumour of Bone (GCTB) receiving a monthly injection of HD-BMAs (7)

Generally, the first group has been associated with a higher risk of MRONJ, ranging from 1% to 20% (8). In the second group, the risk of MRONJ falls within the range of 0.01%–5.2% (9).

In the third group, composed by patients affected by BC or PC without bone metastases and treated with BMAs for the prevention of CTIBL, the incidence of MRONJ was observed between 0% and 10.4% (6, 10–12). Within the limitation of the available evidence, the fourth group falls within the same at-risk category as patients receiving HD-BMAs for bone metastases (1).

To date, oral health preventive measures remain the most effective strategy to reduce MRONJ incidence before and are exercised during, and after the initiation of treatment with medications associated with an increased risk of MRONJ (13, 14). The major goal of MRONJ prevention is to diagnose, treat, and eradicate any oral diseases that are known to increase the MRONJ risk to promote good oral health. Additional targets of primary prevention are enhanced communication between medical and oral health care providers to establish a beneficial interdisciplinary approach to at-risk patients and patient counseling (1). The latter should also make patients aware of the local risk factors of MRONJ and possible clinical manifestations of MRONJ to improve secondary preventive measures. If adequately informed, the patient becomes the primary sentinel for this oral pathology, facilitating early diagnosis of MRONJ and rapid access to treatment.

This systematic review aims to investigate the awareness of MRONJ among patients assuming BMAs.

## 2 Materials and methods

### 2.1 Protocol

A systematic literature search was conducted independently by two authors (RM and MC). The protocol for this study was designed following the Preferred Reporting Items for Systematic Reviews and Meta-Analyses (PRISMA) guidelines (15).

### 2.2 PICo and research question

The research question was designed based on PICo items in which:

P: patients under BMA and/or AA therapy
I: evaluation of knowledge about the risk of MRONJ
Co: worldwide

The systematic review was based on the following research question: Do patients at risk of MRONJ know the risk about MRONJ onset?

### 2.3 Data sources and search strategy

A selection of studies concerning the awareness of patients of the risk of MRONJ was performed.

Records were identified using different search engines (i.e., Medline/PubMed, Scopus, Web of Science) and by scanning references lists of articles.

For the search strategy, MeSH terms and free text words were combined through Boolean operators as follow: (Bisphosphonate-Associated Osteonecrosis of the Jaw OR Medication-related Osteonecrosis of the Jaw OR BRONJ OR ONJ OR MRONJ OR ARONJ OR DRONJ) AND (Awareness OR knowledge OR Informed consent OR information OR perception OR attitude). Research was completed in January 2024.

### 2.4 Eligibility criteria

The inclusion criteria for the studies were as follows:

− Human studies
− English language
− At least 30 patients for study
− Only study evaluating the patients' awareness of MRONJ

Exclusion criteria were studies focused on other aspects not specifically related to patients' awareness on MRONJ, narrative and systematic reviews and meta-analyses; case reports and studies with less than 30 patients.

### 2.5 Statistical analysis

Selected studies were reviewed to detect outcomes of interest. For each study, the following data were extracted using a pre-designed data extraction Excel sheet. The following parameters were collected:

i. Study characteristics: name of the first author, year of publication, name of the country where the study was performed, study aim.
ii. Patient characteristics: gender, mean age, patients' primary diseases, MRONJ onset.
iii. ONJ-related drug therapy characteristics: ONJ-related drugs, type of BMAs or AAs administration, molecule of BP.
iv. Interview process data: number of questions and duration of interview process.
v. Outcomes: awareness about MRONJ and source of information.

Some data were not present in all the studies included in the review. Continuous variables were summarized with mean values and standard deviations, while categorical variables were expressed as counts and percentages. The questions emerging from the bibliographic research were analyzed through a five-level framework, considering the fundamental questions of “what?”, “who?”, “where?”, “when?” and why?”.

## 3 Results

### 3.1 Screening process and study selection

**Figure 1:**
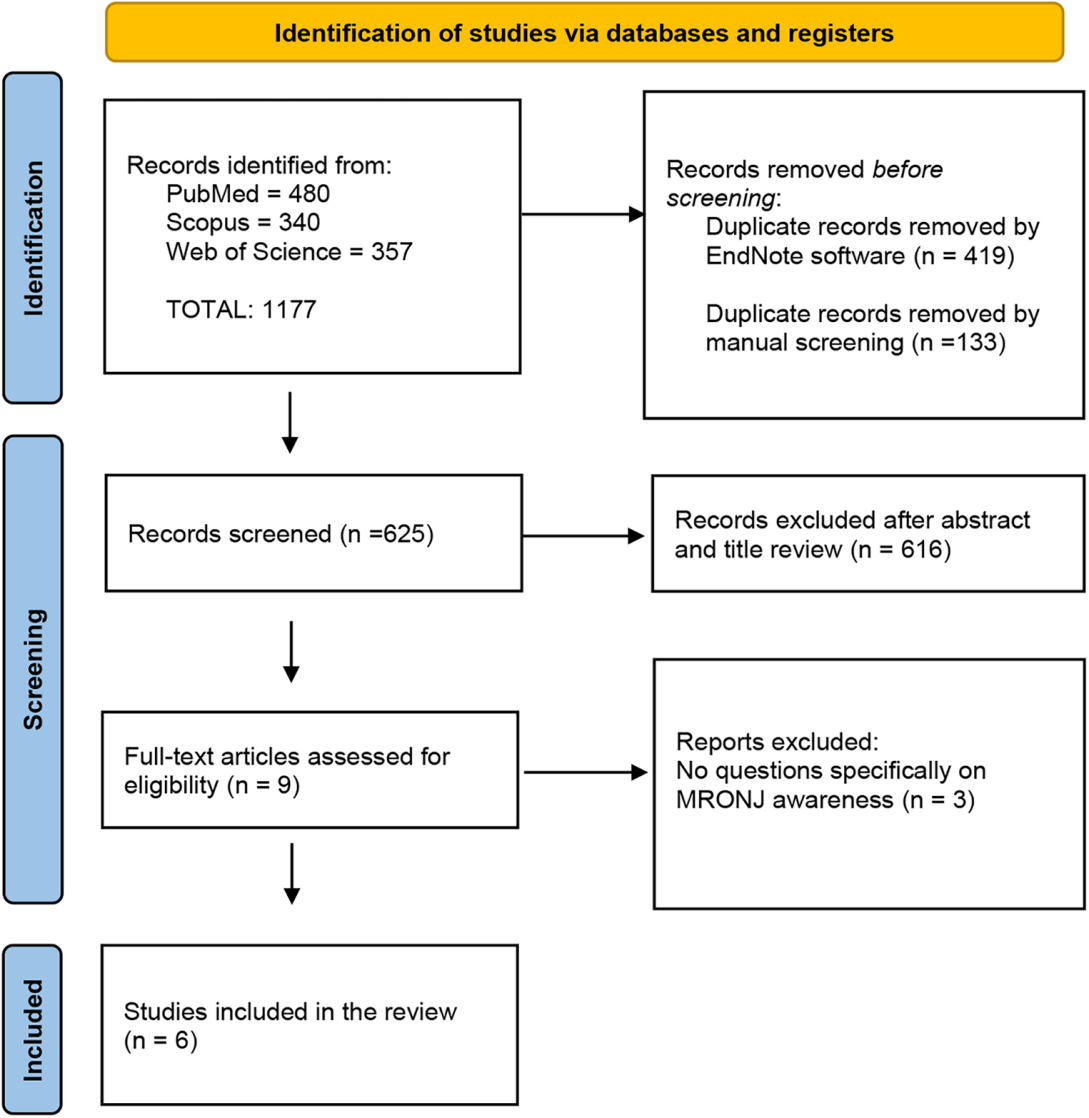
PRISMA 2020 flow diagram.

The initial search strategy identified 1,177 records (PubMed = 480, Scopus = 340, Web of Science = 357). These references were integrated into the EndNote reference software tool (Endnote X9.3.2, Clarivate Analytics). Four hundred and nineteen duplicates were removed by Endnote software and 133 duplicates were removed by manual screening. Then, the screened process of 625 studies was performed based on the title and abstract, and 616 records were excluded. Subsequently, a full-text evaluation of 9 studies was carried out. Finally, 3 records were excluded, and 6 papers were included in the current review. A detailed flow chart of the selection process is provided in Figure 1.

### 3.2 Characteristics of the included studies

The main characteristics of included studies are reported in Table 1. Six studies were included in this review. The studies were published between 2010 and 2022. One study was performed in the USA (16), one in Germany (17), one in Saudi Arabia (18), one in Jordan (19), one in Bulgaria (20) and one in Brazil (21).

**Table 1:** Characteristics of included studies.

First author	Year	Country	Sample size	Male/female	Mean age (range)	Patients’ primary diseases	ONJ-related drugs	BMAs (molecule type)	Awareness about MRONJ	Source of information
Migliorati C.A.	2010	USA	73	2/71	66 (44–88)	Osteoporosis: 54 (74%); Osteopenia: 16 (22%); Breast cancer: 1 (1%); Unknown: 2 (3%)	BPs	alendronate: 44 (60%); risedronate: 21 (29%); ibandronate: 7 (10%); zoledronate: 1 (1%)	13 (18%)	n.d.
Bauer J.S.	2012	Germany	55	8/47	61.9 (45–84)	Osteoporosis: 30 (54.5%); Advanced breast cancer:17 (30.9%); Advanced prostate cancer:8 (14.5%)	BPs	zoledronate: 20 (36.4%); alendronate: 23 (41.8%); risedronate: 8 (14.5%); ibandronato: 9 (16.4%); pamidronate: 1 (1.8%); Etidronic acid: 2 (3.6%); Diphos®: 1 (1.8%); clodronate: 1 (1.8%)	15 (27.3%)	Package insert: 34 (62%); Oncologist: 9 (16%); General practitioner: 7 (13%)
Al Abdullateef A.	2020	Saudi Arabia	68	18/50	20–80	Osteoporosis and cancer patients	BPs, DNB or Aas	n.d.	23 (33.82%)	Physicians: 17 (70.84%); Nurses 1 (4.17%); Dentists 6 (25%)
El-Ma’aita A.	2020	Jordan	110	26/84	40–78	Osteoporosis: 87 (79.1%); Osteopenia: 7 (6.4%); Paget disease: 1 (0.9%); Multiple myeloma: 2 (1.8%); Malignancy: 13 (11.8%)	BPs	n.d.	15 (12.4%)	Physician: 5 (33%); Dentist: 6 (40%); Self-educated: 1 (6.7%); Other sources: 3 (20%)
Hristamyan M.	2022	Bulgaria	112	58/54	68 (38–85)	Breast cancer: 45 (40.18%); Prostate cancer: 40 (35.71%); Multiple myeloma: 7 (6.25%); Lung cancer: 2 (1.79%); Other: 18 (16.07%)	BPs	n.d.	20 (17.86%)	Oncologist: 13 (11.61%); Friend/another patient: 13 (11.61%); Doctor with another specialty:7 (6.25%); Dentist: 3 (2.68%); Internet: 3 (2.68%); Leaflets: 3 (2.68%); General Practitioner: 1 (0.89%)
de Lima-Souza R.A.	2022	Brazil	65	6/59	65.2 (42–82)	Osteoporosis: 59 (90.8%); Breast cancer: 3 (4.6%); Lung cancer: 1 (1.5%); Multiple myeloma 2 (3.1%)	BPs	alendronate: 57 (87.7%); zoledronate: 10 (15.4%); risedronate: 9 (13.8%); ibandronate 2 (3.1%).	6 (9.2%)	Dentist: 2 (3.1%); Physicians: 2 (3.1%); Other (television): 2 (3.1%)

In total, 483 patients were evaluated, of which 118 were males and 365 were females (24.4% vs. 75.6%, respectively) with an age ranging from 20 to 88 years.

Regarding the primary disease, 254 patients were affected by osteometabolic diseases (254/483, 52.6%), 141 patients were affected by cancer (141/483, 29.2%), and 20 patients were affected by “other” or “unknown” diseases (20/483, 4.1%). In one study, osteoporosis and cancer patients were included but it was not specified how many patients were affected by cancer and how many patients were affected by osteoporosis, respectively (18).

In detail, among patients affected by osteometabolic diseases, 230 were affected by osteoporosis (230/254, 90.5%), 23 were affected by osteopenia (23/254, 9.1%) and 1 patient was affected by Paget disease (1/254, 0.4%). Among cancer patients, 66 were affected by breast cancer (66/141, 46.8%), 48 were affected by prostate cancer (48/141, 34%), 13 were affected by “malignancy” (13/141, 9.2%), 11 were affected by multiple myeloma (11/141, 7.8%), and 3 patients were affected by lung cancer (3/141, 2.1%).

In five studies, patients undergoing BP therapy (16, 17, 19–21) and in one study patients undergoing BP, DNB and/or AAs therapy (18).

Regarding the specific BP therapy, only 3 studies specified the BP molecule. Based on the available data, 124 patients undergoing alendronate therapy, 38 patients risedronate therapy, 31 patients zoledronate therapy, 18 patients ibandronate therapy, 1 patient pamidronate therapy and 1 clodronate therapy.

Four studies (4/6, 66.7%) reported how many questions were included in the interview to assess patients' level of knowledge about the MRONJ: 4, 12, 13 and 21, respectively (16–18, 21), respectively.

Of the 483 included patients, 391 were not aware of the possibility of MRONJ onset (391/483, 81%) and 92 were aware of it (92/483, 19%).

The sources of information were the package insert (34/92, 36.9%), physician (24/92, 26.1%), oncologist (22/92, 23.9%), dentist (17/92, 18.5%), friend or another patient (13/92, 14.1%), general practitioner (8/92, 8.7%), doctor with another specialist (7/92, 7.6%). Other sources were television (5/92, 5.4%), internet (3/92, 3.3%), leaflets (3/92, 3.3%), nurses (1/92, 1.1%) and “self-education” (1/92, 1.1%) (17–21). In one study, the source of information was not described (16).

## 4 Discussion

MRONJ is a drug adverse reaction that can greatlsy affect the quality of life of patients if not promptly diagnosed and treated (6).

To the best of our knowledge, very few studies have investigated patient awareness of MRONJ, and, to date, this is the first systematic review on the topic.

The issues that arose from the literature search can be analyzed through a five layered framework, considering the basic questions of “*what?”*, “*who?*”, “*where?*”, “*when?*” and “*why?*”. Once completed this scheme, the question of “*how?*” will be addressed, evaluating the possible remedies to the identified problems.

Regarding the first point (*what?*), the selected studies revealed a lack of awareness concerning the risks associated with MRONJ (81%). This issue has a twofold dimension: on the one hand, patients' lack of awareness of the risks related to MRONJ and its impact on compliance levels; on the other hand, the inadequate level of communication on behalf of healthcare professionals involved, as related to their informative duty. Informing the patient of any adverse drug events is not only an ethical duty but also a legal responsibility of the prescribing physician and healthcare professionals (22). Just as informed consent is obtained before performing a surgical procedure (e.g., tooth extraction or dental implant surgery), healthcare professionals should also take the time to explain and inform the patient of the potential benefits and risks associated with drug therapy before prescribing it (23).

The present systematic review shows that only 19% of patients undergoing BMA therapy were aware of the possibility of MRONJ onset, highlighting poor communication of this adverse reaction by health specialists and consequently poor participation in prevention programs by patients.

The second point of this analysis (*who?*) concerns the role of the different health professionals who take part in the therapeutic process related to the manifestation of MRONJ.

Regarding the source of information, in the present study, most patients learned about MRONJ from the package insert (36.9%), followed by health specialists, friends or other patients (14.1%), television (5.4%), the internet (3.3%), and leaflets (3.3%). Among health specialists, those who most informed patients about the risk of MRONJ were physicians (26.1%), followed by oncologists (23.9%), dentists (18.5%), and general practitioners (8.7%).

Patients at risk of MRONJ are generally managed by a multidisciplinary team comprising the prescribing physician (e.g., bone experts, oncologist), oral health care specialists, and pharmacist.

Dentists play a fundamental role in the prevention of MRONJ as they can control local risk factors, which are modifiable, including dental, periodontal, and periapical infections, ill-fitting prostheses, dentoalveolar, and dental implant surgery. If patients are adequately informed about the potential risks of BMA therapy and the important role of periodic follow-up visits, the incidence of MRONJ could be reduced and the early diagnosis could be improved.

In some countries, the multidisciplinary team would also include nurses. According to a recent systematic review, nurses can play a pivotal role in facilitating multi-professional management of MRONJ by communicating with patients to ensure compliance with preventative measures, and with patients' physicians and dentists to ensure early detection and referral for prompt treatment of MRONJ (24).

So, in a multidisciplinary context marked by the importance of preventive measures, the interaction between the different professionals who relate with the patient becomes a central aspect for the successful outcome of medical treatments and increases the patient's quality of life (25). Data emerging from our study are alarming as they demonstrate that less than a third of patients had been informed by doctors of the risk of MRONJ; this could be attributable to a lack of doctor awareness of MRONJ or doctors' dismissal of the possibility of this complication. In this sense, lack of training, informational and communicative failures fall within the liability sphere of each professional, not only in strictly legal terms, but also in a broader sense, having to do with the ethical dimension of the health professions. Therefore, the lack of patients' awareness with respect to MRONJ risks must be considered in the light of this interaction, paying attention to the communication quality not only in the doctor-patient relationship but also from an interprofessional perspective (26).

The interprofessional dimension is closely related to the following level of analysis (*where?*), which concerns the setting where the patient receives relevant information about MRONJ risks. The multidisciplinary nature of the clinical environment leads to the multiplication and fragmentation of medical information, with the result that the patient has to gather and process different sources of information without there being a comprehensive source from which to obtain a complete picture of the issue at hand. Each healthcare professional has an informational duty, but the fragmentation of its fulfillment brings about the risk of losing sight of the final goal, which is enabling the patient to fully understand the drug therapy effects.

Similarly, the chronological aspect of the therapeutic intervention (*when?*) can affect the fulfillment of the duty of information in its temporal development. In fact, the intervention of different healthcare professionals takes place at different times in the therapeutic process, depending on the clinical and pathological course of each patient. Regarding the prevention of MRONJ, to date, oral health primary preventive measures remain the most effective strategy to prevent it before, during, and after the initiation of BMA therapy (1). Owosho AA et al. emphasized the need for oral evaluation before commencing therapy with BMA. In their study they compared patients who underwent a dental examination before the start of BMA therapy (group I) with patients who underwent a dental examination after the start of BMA therapy (group II); the result was that in group I, 0.9% of patients developed MRONJ, while in group II 10.5% (14). Ideally, all patients at risk of MRONJ should be subjected to dental examination before the BMAs administration and periodic follow-up visits during and after it. For cancer patients with BM or multiple myeloma, there are guidelines and national recommendations regarding the need of a dental visit before the start of BMA therapy. The same does not exist for the patients affected by osteometabolic diseases, so although the risk of developing MRONJ is lower, a visit within the first six months of starting therapy is still suggested (27). Furthermore, the patients assuming BMAs should undergo periodic dental visits to improve the MRONJ diagnosis in the early stage. Generally, dental check-ups should be every four months for cancer patients with BM or multiple myeloma while every six months for patients affected by osteometabolic diseases, unless periodontitis is present (28). It is important to specify that the multidisciplinary approach does not reduce the responsibility of the individual professional, but rather forces us to frame the patient differently, working as a team. Therefore, the responsibility of informing the patient about the risk of MRONJ is not assigned exclusively to a single professional but rather distributed among healthcare professionals of the team throughout the patient's life. Another important aspect not to be underestimated is the fragility of this category of patients, often affected by multiple diseases and undergoing different drug therapies. So, the clinician should heal or, in the most difficult cases, accompany the patient during her illness, aiming to prevent further complications that could worsen her quality of life.

Once the issues of “*what?”*, “*who?*”, “*where?*”, “*when?*” have been examined, the analysis must focus on the causal factors (“*why?*”). These factors can be approached from a dual perspective, considering both the professional conduct and how it impacts patients' awareness. In fact, the lack of adequate training and collaboration of healthcare professionals has showed to influence the duty to inform the patient, thus affecting the levels of patient awareness. Concerning the medical personnel involved in MRONJ prevention, a defect in training or continuing education combines with the insufficiency of collaborative and interprofessional practices. An atomistic outlook and a rigid delimitation of individual liability result in the loss of the collective dimension of healthcare, which implies a collaborative approach to patient care (29). The unfulfillment of informational duty and the inefficacy of interprofessional collaboration add up to further communicative problems that may occur at the individual stages of the therapeutic process, in the relationship between practitioner and patient. Among the factors that may affect the efficiency of communication there is, for example, the reticent attitude of physicians about the adverse effects of the drug therapy, due to the fear that it may negatively influence patient compliance (30). Professionals should therefore assume a communicative style that, considering the risks that drugs produce, could convince the patient to follow the prevention protocol and provide the necessary measures to intervene promptly at the first manifestation of adverse reactions. Another important aspect is that if the patient is not correctly informed and does not feel an active part in the therapeutic process, she does not trust, running the risk of interrupting the treatment. An aspect that should not be underestimated is also that linked to the gender difference, as in the present study the patients included are mostly females (75.6%). In a recent study, it has been reported that women exhibit more positive attitudes about dental visits, have greater oral health literacy, and demonstrate better oral health behaviors than men (31).

Patients' awareness of MRONJ risks stems in part from communication and collaboration problems among the healthcare professionals, in addition to other factors such as patients' health literacy and their capability of understanding therapeutic indications. Instead, the low level of patient awareness is related to a fragmented view of the context of care, which fails to grasp the relational dimension of informational and communicative dynamics (32).

Once completed the initial framework of analysis (“*what?”*, “*who?*”, “*where?*”, “*when?*” and “*why?*”), it is necessary to consider the available strategies and tools to address the lack of awareness of MRONJ risks (*how?*). In the first place, this issue needs to be addressed through the implementation of training and continuing education programs for healthcare professionals.

Integrating information on MRONJ risk into routine patient consultations and training healthcare professionals to communicate the risks associated with BMAs effectively could improve patients’ knowledge of this adverse effect, thereby promoting prevention and reducing the incidence of MRONJ such an approach is necessary but not sufficient to address all the criticalities that emerged from the present study. There is a need to overcome the fragmented vision of healthcare professions and their respective duties, moving away from an individualistic view of liability and deontological duties (33). Indeed, the element that brings together different healthcare professions is the duty of care toward the patient, which can work as a tool for rethinking interprofessional dynamics and promoting an authentically patient-centered therapeutic model. In a clinical scenario marked by a strong multidisciplinary approach such as the one considered in this study, teamwork is crucial for the success of the therapy, from the stage of prevention to diagnosis and to the monitoring of treatment outcomes. In this sense, a type of training that is not only focused on scientific knowledge but also on interprofessional collaboration should be promoted (34, 35). Teamworking should start from the communication with the patient, continuing up to the decision-making phase, in which a shared perspective should not be limited to the relationship between practitioner and patient, but should also shape the relationship between the healthcare professionals themselves. In this sense, the concept of care allows to overcome the lack of awareness, combining the centrality of meeting the patients' needs with the importance of collaboration among health professionals (“patient-centred collaborative care”). Ultimately, alongside an increased focus on targeted training programs concerning MRONJ, the promotion of relational and collaborative care is also likely to have a positive impact on patients' awareness, consequently increasing the margins of compliance and adherence to therapy.

This review possesses some limitations derived from the heterogeneity of the included studies.

In detail, the studies were based on different questionnaires composed of different items, which makes the studies not exactly comparable, they gave us a general overview for future studies. Moreover, the studies analyzed different categories of patients at risk of MRONJ, including patients affected by osteometabolic diseases and cancer. Future studies could analyze specific MRONJ risk categories, and possibly confirm the generalizability of the findings of the present systematic review.

## 5 Conclusions

To date, preventive strategies are considered the most appropriate approach for reducing the risk of MRONJ in patients who are candidates for ONJ-related drugs, during and after treatment. The patient must be fully aware of the possibility of adverse reactions to the drug, local risk factors and the importance of follow-up visits, which implies effective and exhaustive communication by each multidisciplinary team member.

## Data Availability

The raw data supporting the conclusions of this article will be made available by the authors, without undue reservation.
